# Non-pharmaceutical interventions restructured the upper respiratory bacterial microbiota in children under 2 during COVID-19: implications for infection control strategies

**DOI:** 10.3389/fmicb.2026.1737843

**Published:** 2026-02-09

**Authors:** Yufeng Wang, Wenxin Shen, Lina Xu, Jiaoyang Li, Xuena Xu, Suqing Chen, Chuangli Hao, Wujun Jiang

**Affiliations:** 1 Department of Respiratory Medicine, Children's Hospital of Soochow University, Suzhou, China; 2 Department of Pediatrics, The Affiliated Suqian First People's Hospital of Nanjing Medical University, Suqian, China

**Keywords:** 16S-rDNA analysis, children, COVID-19, lower respiratory tract infections, upper respiratory tract microbiota

## Abstract

The COVID-19 pandemic substantially altered pediatric respiratory infection patterns. This study assessed the impact of non-pharmaceutical interventions (NPIs) on upper respiratory bacterial epidemiology and microbiota composition in children under 2 years hospitalized with lower respiratory tract infections (LRTIs). Clinical data from 24,159 children admitted between January 2019 and December 2020 were retrospectively analyzed. Following NPI implementation, the overall culture-based bacterial detection rate declined from 61.02% to 18.38%. In an RSV-positive subgroup (pre-COVID-19, *n* = 95; COVID-19, *n* = 118), upper respiratory microbiota profiles were characterized using 16S rRNA gene sequencing. Alpha diversity increased significantly, while beta diversity showed distinct community separation between periods (Bray–Curtis distance, PERMANOVA *P* = 0.01). Taxonomic shifts included increased *Proteobacteria* and *Actinobacteria* and reduced *Firmicutes*, along with decreased *Streptococcus* and enrichment of *Rothia, Dolosigranulum*, and *Corynebacterium*. Overall, NPIs implemented during the COVID-19 pandemic were associated with marked alterations in the upper respiratory bacterial microbiota of RSV-positive young children, highlighting potential implications for future pediatric infection control strategies.

## 1 Introduction

Lower respiratory tract infections (LRTIs) are among the most common infectious diseases in children and represent a leading cause of morbidity and mortality in hospitalized children under 5 years of age worldwide (GBD 2021 Lower Respiratory Infections Antimicrobial Resistance Collaborators, 2024). Respiratory infections are caused by a wide spectrum of pathogens, including bacteria, viruses, and atypical microorganisms, with bacterial pathogens playing an important role either as primary agents or through co-infection. Epidemiological data from China indicate that between 2009 and 2019, 22.8% of patients with acute respiratory infections tested positive for at least one bacterial pathogen, with the highest detection rates observed in young children; notably, the bacterial positivity rate in children under 5 years reached 23.9% (Li et al., 2021). Growing evidence suggests that interactions between the respiratory microbiota and the host immune system critically influence the onset, progression, and outcomes of respiratory diseases. Disruption of the normal respiratory microbial community may facilitate the overgrowth of potential pathogens, thereby increasing susceptibility to LRTIs (García-Rodríguez and Fresnadillo Martínez, 2002).

In December 2019, the emergence of severe acute respiratory syndrome coronavirus 2 (SARS-CoV-2) in Wuhan, China, triggered the global coronavirus disease 2019 (COVID-19) pandemic (Guan et al., 2020). In response, the World Health Organization declared COVID-19 a global pandemic on March 11, 2020 (Cucinotta and Vanelli, 2020). China subsequently implemented extensive non-pharmaceutical interventions (NPIs), including lockdowns, social distancing, school closures, and mandatory mask use, as part of a nationwide zero-COVID strategy (Fricke et al., 2021). These interventions effectively reduced the transmission of SARS-CoV-2 and other respiratory viruses, while also reshaping the epidemiology of respiratory bacterial infections (Xu et al., 2024). In parallel, changes in healthcare-seeking behavior and reductions in antibiotic use during the pandemic may have further influenced bacterial circulation and colonization patterns (Mamun et al., 2021; Friedli et al., 2022). Interestingly, children generally exhibited lower susceptibility to SARS-CoV-2 infection and milder clinical manifestations compared with adults, although the mechanisms underlying these age-related differences remain incompletely understood (Viner et al., 2021).

The upper respiratory tract microbiota undergoes dynamic development during early childhood and plays a crucial role in shaping mucosal immune responses and host defense against respiratory pathogens (Biesbroek et al., 2014). Previous studies have demonstrated that upper airway microbiota composition is associated with susceptibility to viral respiratory infections, including influenza (Tsang et al., 2020). More recently, respiratory syncytial virus (RSV) infection in infants has been linked to distinct upper airway microbiota profiles, and dominance of genera such as *Streptococcus* or *Haemophilus* has been associated with increased disease severity and adverse clinical outcomes (de Steenhuijsen Piters et al., 2015; Hasegawa et al., 2016). Despite these advances, evidence remains limited regarding how large-scale public health interventions, such as NPIs, influence the upper respiratory microbiota specifically in young children with RSV infection.

Therefore, the present study retrospectively analyzed children under 2 years of age hospitalized with LRTIs in the Suzhou region to characterize changes in bacterial epidemiology before and during the COVID-19 pandemic. In addition, we focused on RSV-positive children to investigate alterations in upper respiratory tract microbiota composition associated with pandemic-related NPIs. By integrating large-scale epidemiological data with microbiota profiling, this study aims to provide insights into how public health interventions reshape the pediatric respiratory microbial ecosystem and to inform future infection control strategies in early childhood.

## 2 Materials and methods

### 2.1 Study population

This retrospective study included 24,159 children aged 28 days to 2 years who were hospitalized with lower respiratory tract infections (LRTIs) at the Children's Hospital Affiliated to Soochow University between January 2019 and December 2020. Patients with repeated visits within 1 week, hospital-acquired infections, or ventilator-associated pneumonia were excluded.

**Figure 1:**
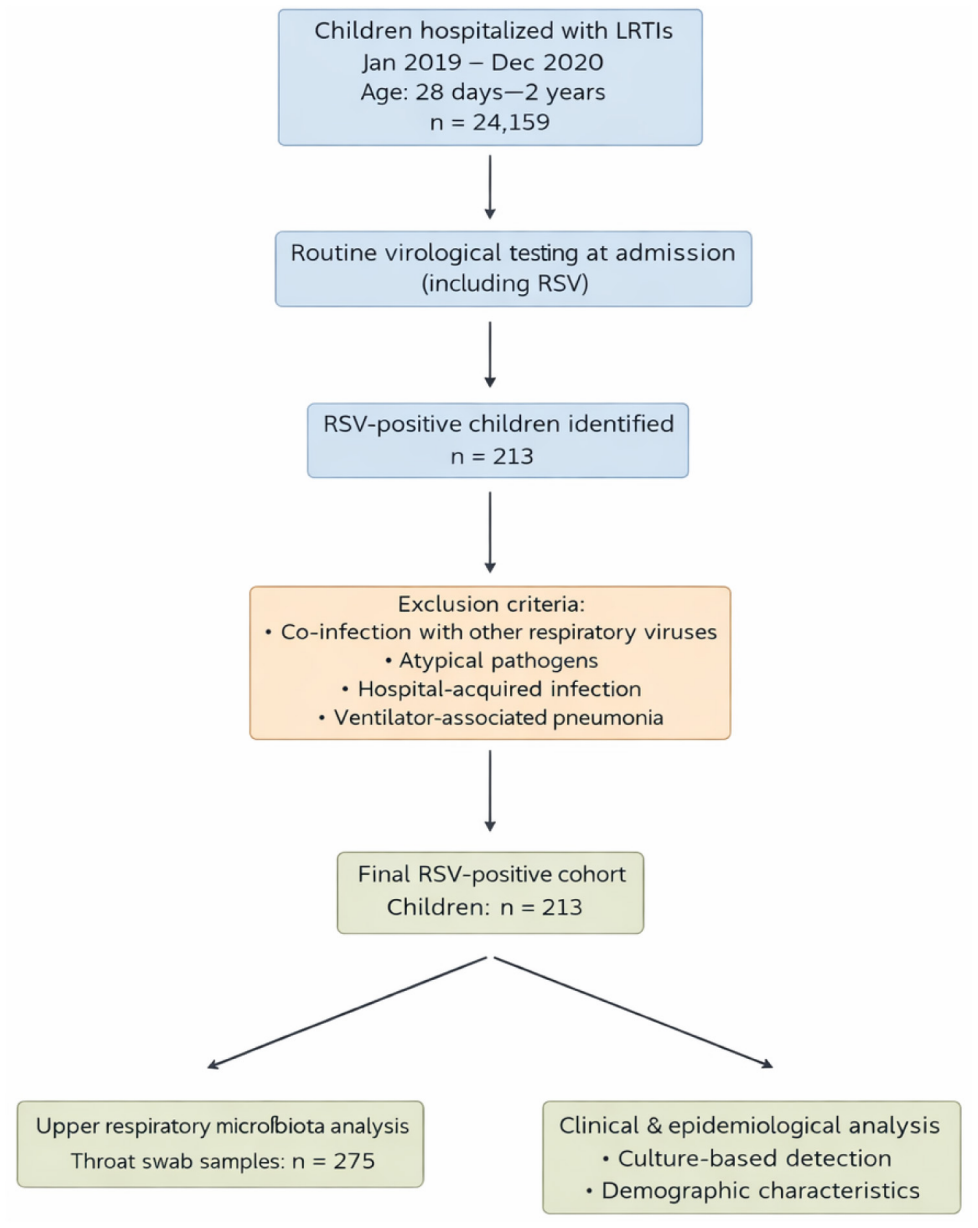
Flowchart of study population selection and RSV-positive subgroup definition. A total of 24,159 children younger than 2 years hospitalized with lower respiratory tract infections (LRTIs) between January 2019 and December 2020 were included. Routine virological testing was performed at admission. Children with confirmed respiratory syncytial virus (RSV) infection and without co-infection with other respiratory viruses or atypical pathogens were included in the RSV-positive subgroup (*n* = 213). Throat swab samples from RSV-positive children were used for upper respiratory tract microbiota analysis.

The study period was divided into the pre-COVID-19 period (January–December 2019) and the COVID-19 period (January–December 2020). A flowchart describing patient selection is provided in Figure 1.

Age-based grouping was applied because early childhood represents a critical period of rapid upper respiratory microbiota maturation and immune development, during which microbial composition and susceptibility to respiratory infections vary substantially with age (McCauley et al., 2024).

The four bacterial species (*Streptococcus pneumoniae, Haemophilus influenzae, Moraxella catarrhalis*, and *Staphylococcus aureus*) were selected as representative pathogens due to their high prevalence in pediatric LRTIs and routine inclusion in clinical microbiological surveillance (Bline et al., 2025).

The protocol of this study was approved by the Ethics Committee of the Children's Hospital of Soochow University (ethical approval no. 2023CS357) and was in line with the Declaration of Helsinki (as revised in 2013). Written informed consent was obtained from at least one guardian of each patient before enrollment. The data from patients were analyzed anonymously.

### 2.2 Sample collection and pathogen detection

Nasopharyngeal aspirates were collected from all enrolled children within 24 h of admission. Detection of common bacterial pathogens (*Streptococcus pneumoniae, Staphylococcus aureus, Haemophilus influenzae, Moraxella catarrhalis*, and *Klebsiella pneumoniae*) was performed using routine clinical culture-based methods combined with biochemical identification as part of standard hospital surveillance.

RSV infection was confirmed by routine clinical virological testing at admission. Children included in the RSV subgroup tested positive for RSV and had no documented co-infection with other respiratory viruses at the time of sampling. Throat swabs were additionally collected from RSV-positive children for microbiota analysis, and the RSV-positive cohort was analyzed separately to avoid confounding with general LRTI microbiological data.

All samples were immediately placed in cryopreservation containers after collection, stored at −80 °C within 1 hour, transported on dry ice, and maintained at −80 °C until further processing for DNA extraction and 16S rRNA gene sequencing. The RSV samples used for sequencing were obtained as part of routine respiratory infection surveillance conducted between 2019 and 2020. Some RSV-positive children contributed more than one throat swab sample during hospitalization; therefore, the number of samples exceeded the number of individuals.

### 2.3 Bacteria DNA extraction, 16S amplification and deep sequencing

The V3–V4 hypervariable regions of the bacterial 16S rRNA gene were amplified using standard primers and sequenced on the Illumina MiSeq platform with paired-end reads. Genomic DNA was extracted using the HiPure Tissue DNA Mini Kit (D3121-02, Magen, Shanghai, China).

Raw paired-end reads were merged using overlap information and processed using the DADA2 pipeline implemented in QIIME 2 (version 2020.6). Reads were quality filtered, denoised, dereplicated, and checked for chimeras to infer amplicon sequence variants (ASVs) without clustering. Rarefaction curves based on observed ASVs were generated to assess sequencing depth and sampling adequacy.

### 2.4 Bioinformatics analysis and statistical analysis

Taxonomic assignment of amplicon sequence variants (ASVs) was performed using the SILVA reference database. Alpha diversity indices, including Shannon and Chao1, were calculated based on the ASV feature table. Beta diversity was assessed using Bray–Curtis distances, and differences in community structure between groups were evaluated using permutational multivariate analysis of variance (PERMANOVA). Differential abundance analysis was conducted using linear discriminant analysis effect size (LEfSe) to identify taxa characterizing each period within the RSV-positive subgroup. Statistical analyses were performed using SPSS version 27.0 (IBM Corp., Armonk, NY, USA), and a two-sided *P* value < 0.05 was considered statistically significant.

## 3 Results

### 3.1 General clinical characteristics

**Figure 2:**
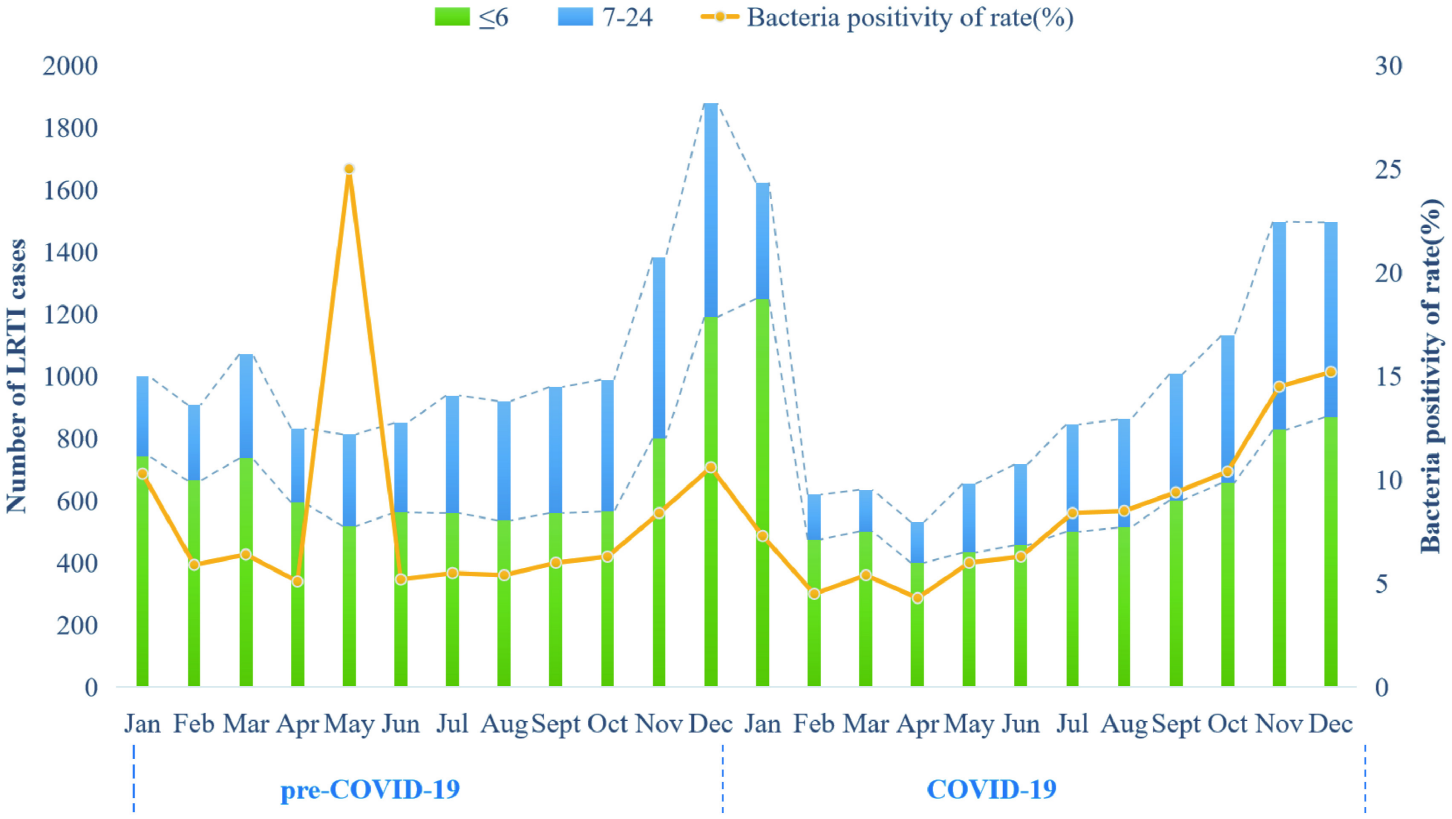
Monthly reported cases of lower respiratory tract infections, monthly positive rates of pathogenic bacteria, and age distribution during the study period from January 2019 to December 2020 in Suzhou. On January 24, 2020, China implemented a nationwide lockdown and a Level-I public health emergency response with non-pharmaceutical interventions. According to the adjustment of Suzhou's COVID-19 policies, we defined two periods from January 2019 to December 2020: from January 2019 to December 2019 (pre-COVID-19), and from January 2020 to December 2020 (COVID-19).

A total of 24,159 children aged 28 days to 2 years hospitalized with LRTIs were included in this study. The overall number of hospitalizations and bacterial detection rates differed markedly between the pre-COVID-19 and COVID-19 periods (Figure 2), as summarized in Table 1.

**Table 1:** Demographic characteristics and bacterial detection rates among children hospitalized for lower respiratory tract infections before and during the COVID-19 pandemic.

**Demographics**	**Total**	**Pre-COVID-19**	**COVID-19**	**χ^2^**	***P***
LRTIS, *n* (%)	24,159	12,546 (51.93)	11,613 (48.07)		
**Gendern**, ***n*** **(%)**				6.87	0.01
Male	14,978 (62.00)	7,877 (62.78)	7,101 (61.15)		
Female	9,181 (38.00)	4,669 (37.22)	4,512 (38.85)		
**Age distribution**, ***n*** **(%)**				0.47	0.50
≤ 6 m	15,506 (64.18)	8,027 (64.00)	7,479 (64.40)		
7–24 m	8,653 (35.82)	4,519 (36.02)	4,134 (35.60)		
**Season**, ***n*** **(%)**					
Spring (3 m−5 m)	4,536 (18.78)	2,716 (21.65)	1,820 (15.67)	140.84	< 0.001
Summer (6 m−8 m)	5,130 (21.23)	2,708 (21.59)	2,422 (20.86)	1.87	0.17
Autumn (9 m−11 m)	6,970 (28.85)	3,336 (26.60)	3,634 (31.29)	64.74	< 0.001
Winter (12 m−2 m)	7,523 (31.14)	3,786 (30.18)	3,737 (32.18)	11.19	< 0.001
Total positive cases^a^, *n* (%)	9,791 (40.53)	7,656 (61.02)	2,135 (18.38)	4,549.01	< 0.001
**Bacteria detection**, ***n*** **(%)**					
*Streptococcus pneumoniae*	891 (3.69)	528 (4.21)	363 (3.13)	19.90	< 0.001
*Moraxella catarrhalis*	913 (3.80)	125 (1.00)	788 (6.79)	555.83	< 0.001
*Staphylococcus aureus*	881 (3.65)	334 (2.66)	547 (4.71)	71.99	< 0.001
*Haemophilus influenzae*	346 (1.43)	77 (0.61)	269 (2.32)	123.85	< 0.001
Others^b^	6,906 (28.59)	6,668 (53.15)	238 (2.05)	3,810.64	< 0.001

^a^Total positive cases refer to the number of children with lower respiratory tract infections in whom at least one pathogenic bacterium was detected.

^b^Others include Streptococcus viridans, coagulase-negative Staphylococcus, Acinetobacter pittii, Haemophilus haemolyticus, Enterobacter cloacae, Klebsiella oxytoca, Stenotrophomonas maltophilia, Acinetobacter nosocomialis, Streptococcus dysgalactiae, Serratia marcescens, Enterobacter asburiae, Enterococcus spp., Streptococcus pyogenes, Acinetobacter junii, Staphylococcus epidermidis, Moraxella non-liquefaciens, Acinetobacter calcoaceticus, Pseudomonas putida, Acinetobacter radioresistens, Lactobacillus spp., Chryseobacterium indologenes, Citrobacter koseri, Flavobacterium meningosepticum, Burkholderia cepacia, and Pseudomonas fulva.

m, month.

Given that subsequent microbiota analyses focused on children with RSV infection, demographic and clinical characteristics of the RSV-positive subgroup were analyzed separately (Table 2). A total of 213 RSV-positive children were included, comprising 95 cases in the pre-COVID-19 period and 118 cases during the COVID-19 period. Age, sex distribution, length of hospital stay, and oxygen therapy requirements were compared between periods.

**Table 2:** Demographic and clinical characteristics of RSV-positive children.

**Characteristic**	**Pre-COVID-19 RSV (*n* = 95)**	**COVID-19 RSV (*n* = 118)**	***P* value**
Age, months, median (IQR)	3 (2.4–7.5)	8 (4–17)	< 0.001
Male sex, *n* (%)	67 (70.5%)	82 (69.5%)	0.99
Length of hospital stay, days, median (IQR)	7 (6–9)	7 (6–9)	>0.5
Oxygen therapy, *n* (%)	7 (7.4%)	20 (16.9%)	>0.05

Continuous variables were compared using the Mann–Whitney *U* test. Categorical variables were compared using the chi-square test or Fisher's exact test as appropriate. *P* < 0.05 was considered statistically significant.

### 3.2 Overall detection of pathogenic bacteria in LRTIs

As shown in Table 1, the overall bacterial positivity rate decreased from 61.02% (7,656/12,546) pre-COVID-19 to 18.38% (2,135/11,613) during the COVID-19 period (*P* < 0.001). The predominant bacteria in both periods were *Streptococcus pneumoniae, Moraxella catarrhalis, Haemophilus influenzae, and Staphylococcus aureus*. Compared to the pre-pandemic period, *Moraxella catarrhalis* increased from 1.00% to 6.79%, *Staphylococcus aureus* from 2.66% to 4.71%, and *Haemophilus influenzae* from 0.61% to 2.32%, while *Streptococcus pneumoniae* decreased from 4.21% to 3.13% (all *P* < 0.001).

### 3.3 Age and seasonal distribution of bacterial pathogens

**Figure 3:**
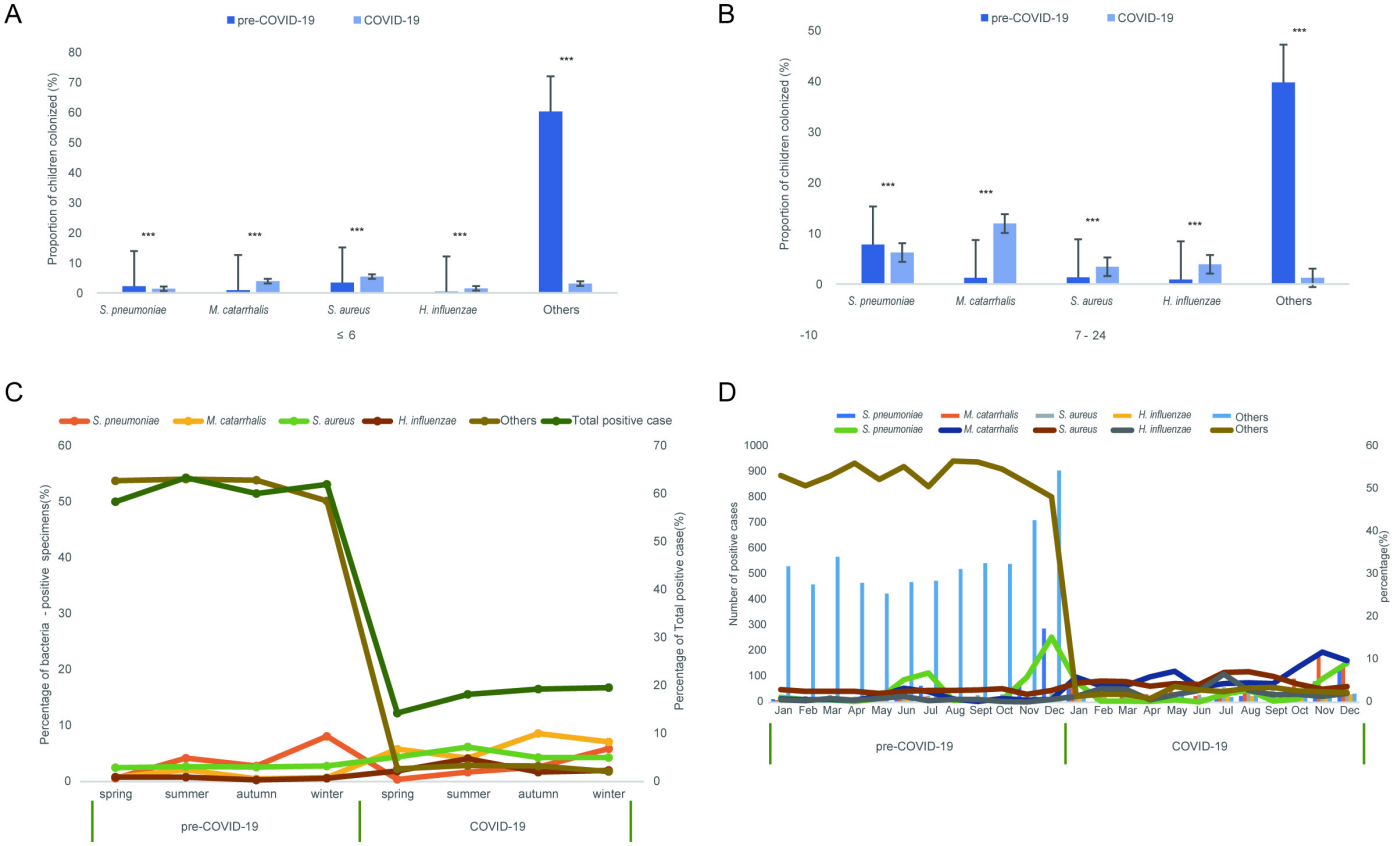
Detection of various pathogenic bacteria distributed by age, season and month before and during the epidemic. **(A, B)** Proportion of children colonized with major respiratory bacteria in the pre-COVID-19 and COVID-19 periods by age (**A**, ≤ 6 months; **B**, 7–24 months). **(C)** Seasonal distribution of bacteria-positive specimens and total positive cases. **(D)** Monthly number and percentage of bacteria-positive cases across the study period. Data are shown as mean ± SEM. ***P < 0.001.

The distribution patterns were consistent between children ≤ 6 months and those 7–24 months (Figures 3A, B). In both age groups, Streptococcus pneumoniae detection decreased, whereas *Staphylococcus aureus, Moraxella catarrhalis*, and *Haemophilus influenzae* increased (all *P* < 0.001). Seasonal trends revealed a shift in bacterial dominance, with *Moraxella catarrhalis* and *Haemophilus influenzae* peaking in autumn and summer, respectively, during the pandemic period (Figures 3C, D).

### 3.4 Diversity of the upper respiratory tract microbiota in the RSV-positive subgroup

A total of 275 throat swab samples from 213 RSV-positive children were included in the microbiota analysis, comprising 95 samples collected during the pre-COVID-19 period and 180 samples collected during the COVID-19 period.

After quality filtering and denoising, a total of 20,657,486 high-quality reads were retained across all samples. The average sequencing depth was 75,118 reads per sample, which was sufficient for downstream diversity and taxonomic analyses.

**Figure 4:**
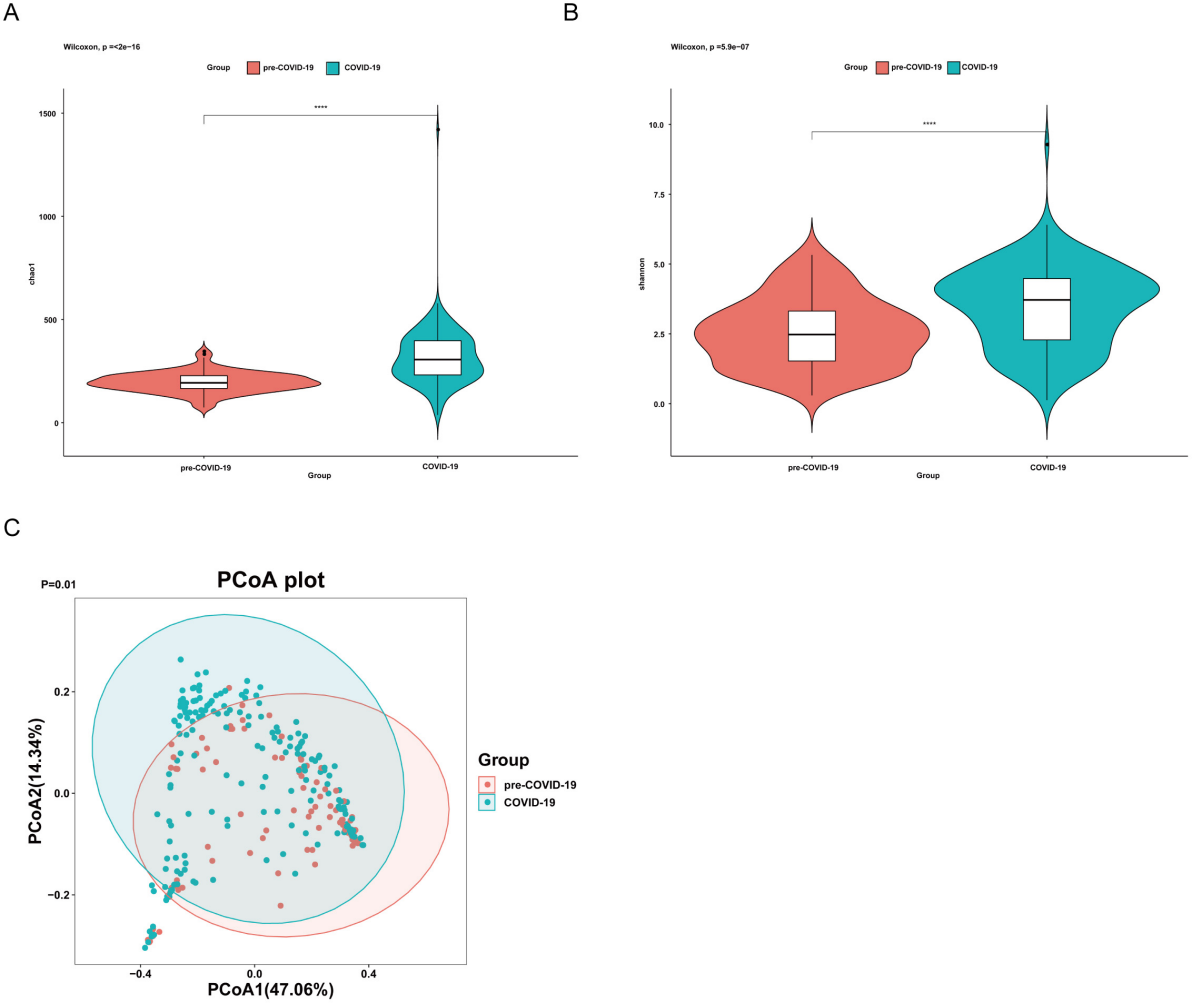
Alpha and beta diversity of the upper respiratory tract microbiota in the RSV-positive subgroup. Alpha diversity was assessed using the Chao1 **(A)** and Shannon **(B)** indices based on throat swab samples from RSV-positive children in the pre-COVID-19 and COVID-19 periods. Beta diversity was evaluated using Bray–Curtis distances and visualized by principal coordinate analysis (PCoA) **(C)**. Each point represents an individual sample, and colors indicate different study groups. Ellipses represent the 95% confidence intervals for each group. Statistical comparisons of alpha diversity indices between groups were performed using the Wilcoxon rank-sum test. *P* < 0.05; *P* < 0.01; ns, not significant.

Rarefaction curve analysis demonstrated that sequencing depth was sufficient for all samples, as the curves reached a clear plateau (Supplementary Figure 1). Alpha diversity was assessed using the Chao1 and Shannon indices. Both indices were higher in samples from the COVID-19 period than in those from the pre-COVID-19 period, indicating increased microbial richness and evenness during the COVID-19 period (*P* < 0.05; Figures 4A, B).

Beta diversity was evaluated using Bray–Curtis distance metrics. Principal coordinate analysis showed separation between samples collected in the pre-COVID-19 and COVID-19 periods (Figure 4C). Permutational multivariate analysis of variance (PERMANOVA) confirmed that overall community composition differed between the two groups (*P* = 0.01).

### 3.5 Composition and differential characteristics of the upper respiratory tract microbiota in the RSV-positive subgroup

Taxonomic profiling of the upper respiratory tract microbiota was performed in the RSV-positive subgroup using the SILVA and NT-16S databases. Relative abundances of the top 30 taxa were visualized using stacked bar plots.

**Figure 5:**
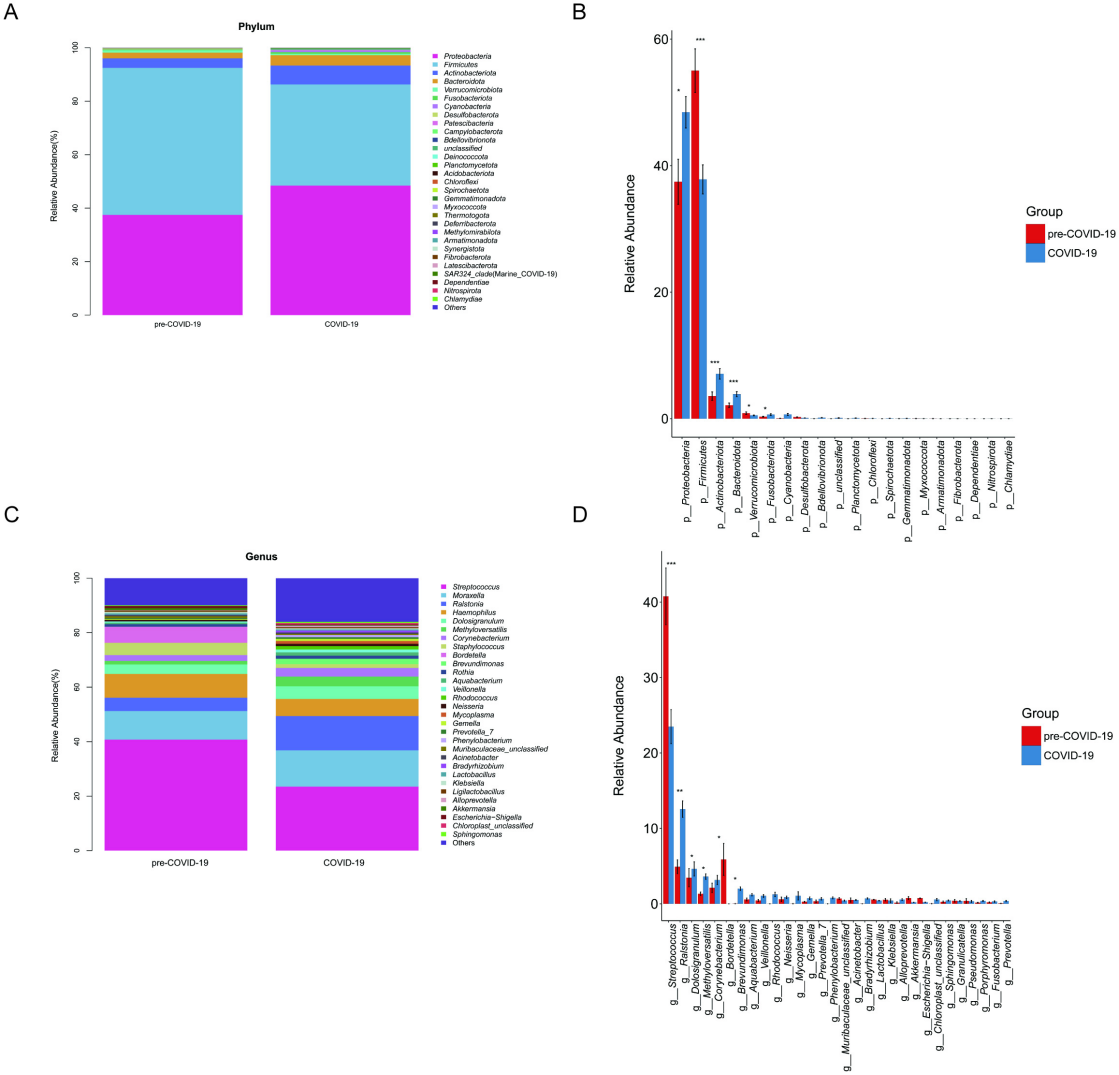
Comparison of upper respiratory tract microbiota composition between the pre-COVID-19 and COVID-19 periods in RSV-positive children. (**A)** Stacked bar plots showing the relative abundance of bacterial phyla in samples collected during the pre-COVID-19 and COVID-19 periods. **(B)** Bar plots depicting the relative abundance of major bacterial phyla, with error bars representing the standard error of the mean (SEM). **(C)** Stacked bar plots illustrating the genus-level microbial composition in the two periods. **(D)** Differential abundance analysis at the genus level between the pre-COVID-19 and COVID-19 groups. Bars represent mean relative abundance ± SEM. Statistical significance between groups was assessed using the Wilcoxon rank-sum test; *P < 0.05, **P < 0.01, ***P < 0.001.

At the phylum level, samples collected during the COVID-19 period exhibited a distinct microbial composition compared with those obtained in the pre-COVID-19 period. Specifically, the relative abundances of *Proteobacteria, Actinobacteriota*, and *Bacteroidota* were increased during the COVID-19 period, whereas *Firmicutes* accounted for a lower proportion (Figures 5A, B).

At the genus level, *Streptococcus* showed a markedly reduced relative abundance in samples collected during the COVID-19 period. In contrast, several genera, including *Rothia, Dolosigranulum*, and *Corynebacterium*, were enriched compared with the pre-COVID-19 period (Figures 5C, D).

Differential abundance analysis based on the Wilcoxon rank-sum test identified 30 taxa that differed significantly between the two periods (*P* < 0.05). Compared with the pre-COVID-19 group, *Streptococcus* and *Bordetella* were significantly depleted in the COVID-19 group, whereas several genera commonly associated with the oral microbiota, including *Alloprevotella* and *Bacillus*, showed increased relative abundances (Figure 5D).

**Figure 6:**
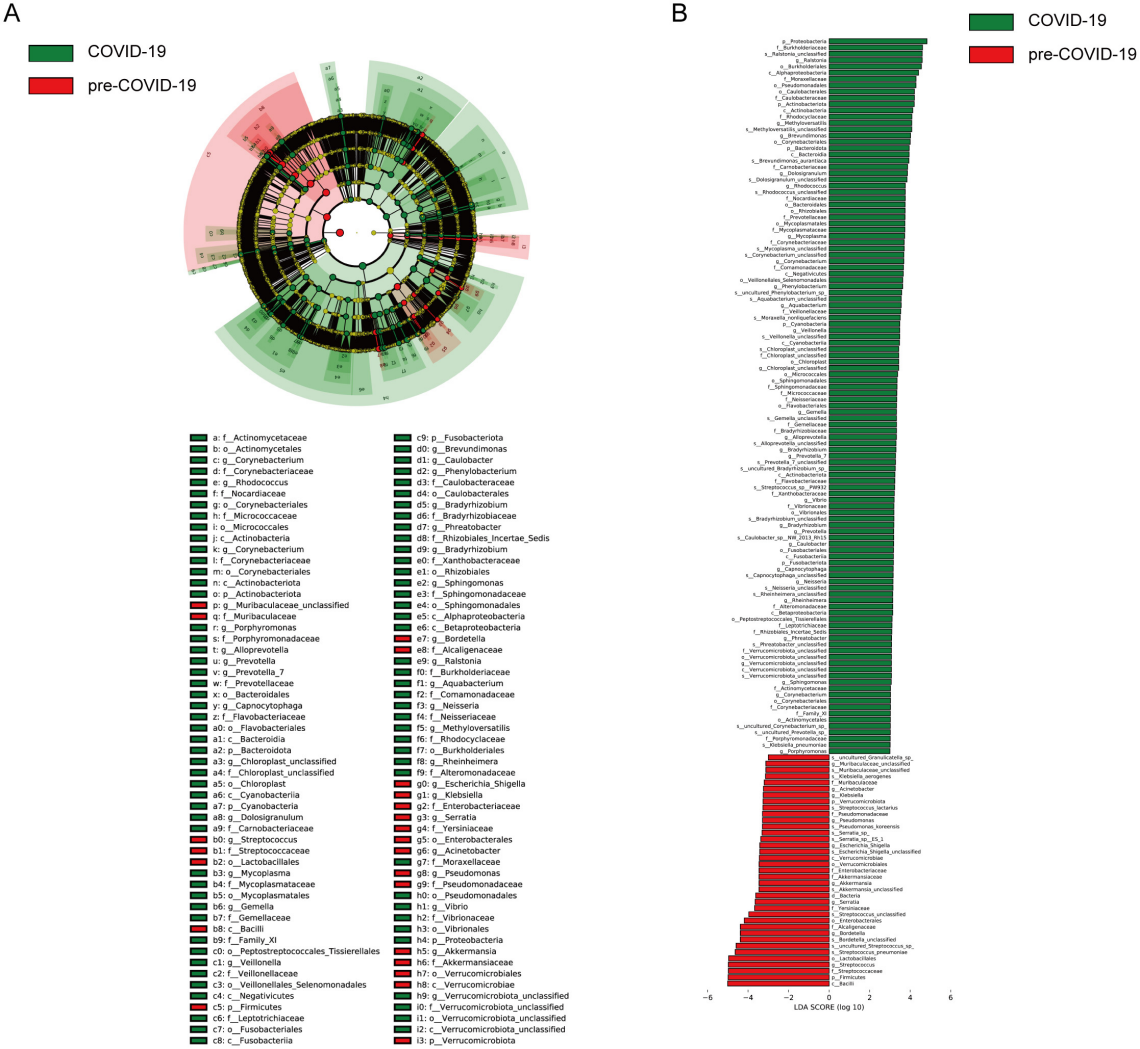
LEfSe analysis of differential taxa in the upper respiratory tract microbiota of the RSV-positive subgroup. **(A)** Cladogram showing phylogenetic relationships of bacterial taxa differentially enriched between the pre-COVID-19 and COVID-19 periods among RSV-positive children. Taxa are arranged hierarchically from phylum to genus from the inner to the outer rings. Red nodes indicate taxa enriched in the COVID-19 group, green nodes indicate taxa enriched in the pre-COVID-19 group, and yellow nodes indicate taxa without significant differences. **(B)** Linear discriminant analysis (LDA) scores identifying taxa with LDA values ≥ 3.0 that discriminate between the two groups. Taxonomic levels are indicated as follows: p, phylum; c, class; o, order; f, family; g, genus.

LEfSe analysis was further applied within the RSV-positive subgroup to identify taxa characterizing each period based on effect size rather than absolute abundance differences. Distinct sets of taxa were identified between the two groups (Figure 6A). During the COVID-19 period, enrichment was observed for *Proteobacteria, Burkholderiaceae*, and *Ralstonia*. At the genus level, *Rothia, Methylobacterium*, and *Brevundimonas* showed higher LDA scores (log10 > 4.0) (Figure 6B).

In contrast, samples from the pre-COVID-19 period were characterized by higher relative abundances of *Firmicutes, Bacilli*, and *Streptococcaceae*, with *Streptococcus* and *Bordetella* remaining predominant genera.

## 4 Discussion

Emerging evidence indicates that non-pharmaceutical interventions (NPIs) implemented to mitigate SARS-CoV-2 transmission have not only controlled COVID-19 but also altered the epidemiological patterns of LRTI-causing bacterial pathogens and the composition of the upper respiratory microbiota (Olwagen et al., 2024; Candel et al., 2023). Children generally exhibit lower susceptibility to SARS-CoV-2 and milder disease courses compared with adults, and pandemic-associated ecological pressures may have distinct effects on the pediatric respiratory microbiota (Hurst et al., 2022). However, data describing microbiota changes in young children during the COVID-19 period remain limited. China's NPIs implemented to control COVID-19 created a population-level barrier against respiratory pathogens and influenced the epidemiological characteristics of LRTI-causing bacteria (Shmueli et al., 2024). In the present study, hospitalizations for LRTIs among children under 2 years of age decreased by 48.07% during the COVID-19 period. Concurrently, the overall bacterial detection rate declined markedly by 42.64%, consistent with reports from other regions in China (Meng et al., 2023; Cai et al., 2022). Together, these findings indicate a pronounced reduction in bacterial LRTI diagnoses during the first year of the COVID-19 pandemic (January–December 2020), highlighting the broad impact of NPIs beyond SARS-CoV-2 containment.

At the pathogen-specific level, we observed a significant reduction in *Streptococcus pneumoniae* detection during the COVID-19 period. This decline likely reflects reduced respiratory droplet transmission and decreased interpersonal contact under NPIs (Brueggemann et al., 2021; Weiser et al., 2018) In contrast, detection rates of *Staphylococcus aureus, Haemophilus influenzae, and Moraxella catarrhalis* increased. These pathogens are common colonizers in early childhood, and their relative increase may reflect the high baseline susceptibility of children under 2 years of age, combined with partial—but not complete—suppression of microbial transmission during the pandemic (Choi and Miller, 2021; Serigstad et al., 2022). This shift suggests that NPIs selectively altered pathogen circulation rather than uniformly suppressing all respiratory bacteria.

Beyond culture-based findings, our microbiota analysis focused specifically on RSV-positive children, allowing us to examine microbial community remodeling within a consistent viral infection background. LEfSe analysis revealed that the pre-COVID-19 RSV subgroup was characterized by enrichment of *Firmicutes*, particularly *Streptococcaceae*, with *Streptococcus and Bordetella* as dominant genera. These taxa have been frequently associated with acute respiratory inflammation and may facilitate bacterial–viral interactions during RSV infection. In contrast, RSV-positive children during the COVID-19 period showed enrichment of *Proteobacteria* and several commensal-associated or low-virulence genera, including *Rothia, Methylobacterium*, and *Brevundimonas*, accompanied by increased microbial diversity.

Previous studies have suggested that commensal-dominated respiratory microbiota profiles, such as those enriched in *Corynebacterium* and *Dolosigranulum*, may be associated with greater microbial stability and reduced susceptibility to viral infections (Man et al., 2017; Mak et al., 1983). Mechanistically, such commensal-enriched profiles have been proposed to influence viral infection outcomes by modulating epithelial barrier integrity, shaping basal innate immune tone (e.g., interferon responsiveness), and limiting secondary bacterial expansion during viral infection (Wirusanti et al., 2022; Ortiz Moyano et al., 2020). Although causality cannot be inferred from the present study, these pathways provide a biologically plausible framework linking microbiota remodeling to viral disease dynamics in early life. In our study, however, both cohorts consisted exclusively of RSV-positive children, indicating that RSV infection itself likely exerted a shared baseline influence on microbial composition. The observed differences therefore more plausibly reflect the combined effects of RSV infection and pandemic-related ecological pressures—such as reduced pathogen exposure and altered social contact—rather than RSV infection alone. This finding underscores the sensitivity of the pediatric upper respiratory microbiota to external environmental and behavioral factors.

Importantly, these results have potential implications for infection control strategies. The reduction in pathogen-dominated microbiota profiles and the shift toward greater microbial diversity during periods of strict NPIs suggest that population-level behavioral interventions can indirectly modulate respiratory microbial ecosystems. In the post-pandemic era, targeted and situational use of infection control measures—such as mask-wearing during peak respiratory virus seasons or in high-risk pediatric settings—may help limit pathogen overgrowth while preserving a more balanced respiratory microbiota. Such microbiota-aware strategies could complement traditional infection prevention approaches, particularly for vulnerable infants and young children.

This study has several limitations. As a single-center retrospective analysis, residual confounding by seasonality, hospitalization timing, or unmeasured clinical variables cannot be fully excluded. Although the culture-based dataset was large, the sequencing cohort was smaller and limited to RSV-positive cases. Additionally, despite inter-batch correction, minor technical variation between sequencing runs may remain. Future longitudinal and multi-omic studies are warranted to clarify the functional consequences of respiratory microbiota remodeling and its role in susceptibility to viral and bacterial respiratory infections.

**Figure 7:**
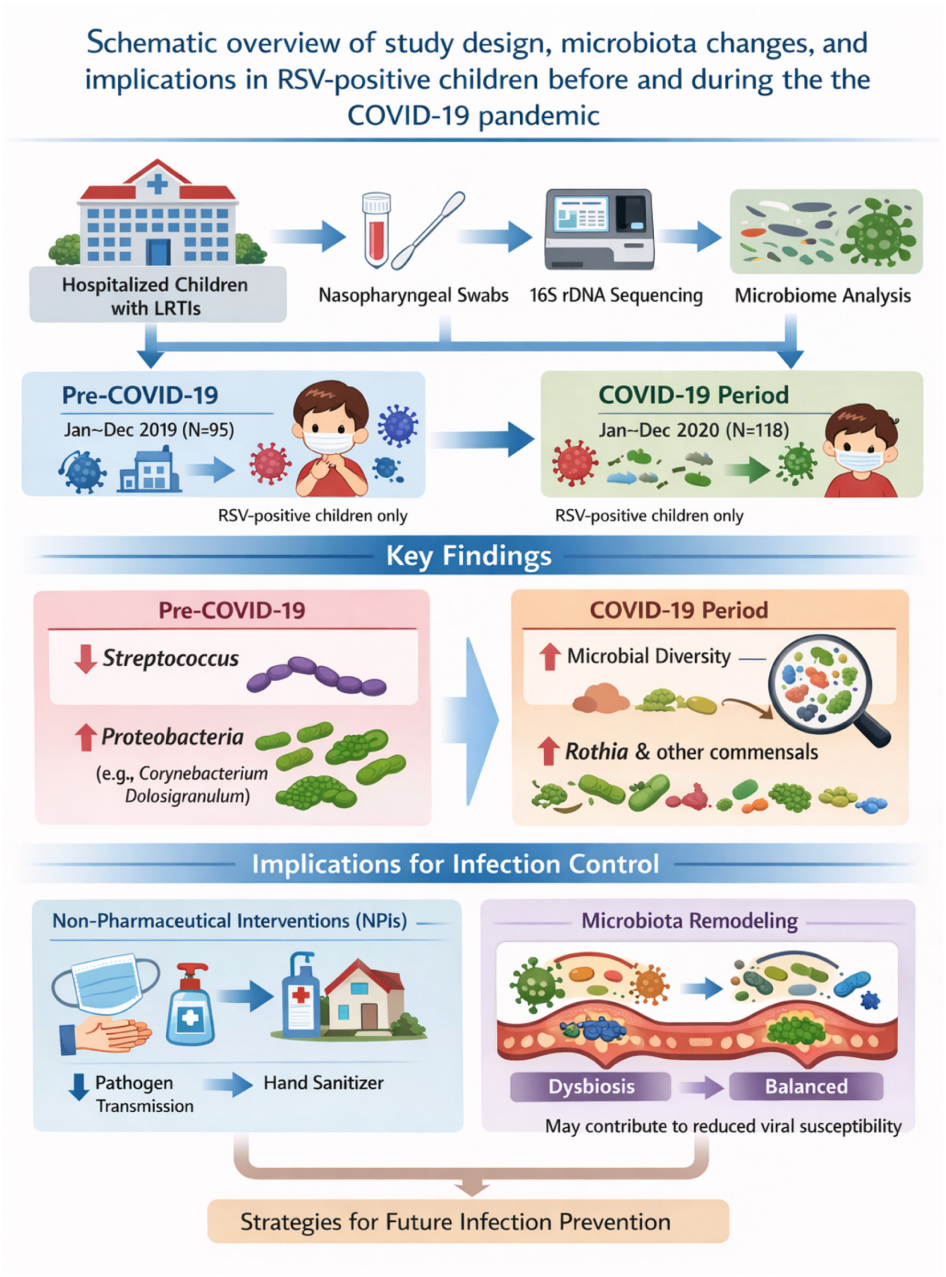
Summary of key findings. This schematic illustration synthesizes the principal findings of the study, highlighting how non-pharmaceutical interventions (NPIs) implemented during the COVID-19 pandemic reshaped the upper respiratory microbiota composition in children with laboratory-confirmed respiratory syncytial virus (RSV) infection. Central observations include a significant reduction in Streptococcus relative abundance and a concomitant increase in alpha diversity–changes that may inform future evidence-based approaches to pediatric infection prevention and microbiota-informed public health strategies.

In conclusion, COVID-19-related NPIs were associated with a substantial reduction in pediatric bacterial LRTIs and significant remodeling of the upper respiratory tract microbiota in RSV-positive children. These findings highlight the indirect but meaningful influence of social and behavioral interventions on respiratory microbial ecology and provide a potential framework for refining infection control strategies in the post-pandemic era (Figure 7).

## Data Availability

The original contributions presented in the study are publicly available. This data can be found here: https://www.ncbi.nlm.nih.gov/, accession number PRJNA1416284.
