# Supplementary material for: Non-pharmaceutical interventions restructured the upper respiratory bacterial microbiota in children under 2 during COVID-19: implications for infection control strategies
Source: Front Microbiol. 2026 Feb 9;17:1737843. doi: 10.3389/fmicb.2026.1737843 (PMC12926401; doi:10.3389/fmicb.2026.1737843)
Supplement: Supplementary file 4 [file Data_Sheet_1.pdf]

Supplementary Table Legends

Supplementary Table 1. Alpha diversity indices of the upper respiratory tract microbiota in the RSV-positive subgroup.

This table presents alpha diversity metrics, including the Chao1 and Shannon indices, calculated for each throat swab sample from the RSV-positive subgroup. Samples are grouped according to the pre-COVID-19 and COVID-19 periods. These data were used for the alpha diversity comparisons shown in Figure 4A and Figure 4B.

Supplementary Table 2. Bray–Curtis distance matrix of the upper respiratory tract microbiota in the RSV-positive subgroup.

This table contains the Bray–Curtis dissimilarity matrix generated from amplicon sequence variant (ASV) relative abundance profiles for all samples in the RSV-positive subgroup. The distance matrix was used for beta diversity analyses, including principal coordinate analysis (PCoA) and permutational multivariate analysis of variance (PERMANOVA), as presented in Figure 4C.

Supplementary Table 3. Differential abundance analysis of upper respiratory tract microbiota between the pre-COVID-19 and COVID-19 periods in the RSV-positive subgroup.

This table provides the raw output of differential abundance analysis comparing microbial taxa between the pre-COVID-19 and COVID-19 periods within the RSV-positive subgroup. The results include taxa with statistically significant differences between groups and were used to generate the stacked bar plots (Figure 5A-B) and to identify discriminative taxa in the LEfSe analysis (Figure 6).

Supplementary Figure

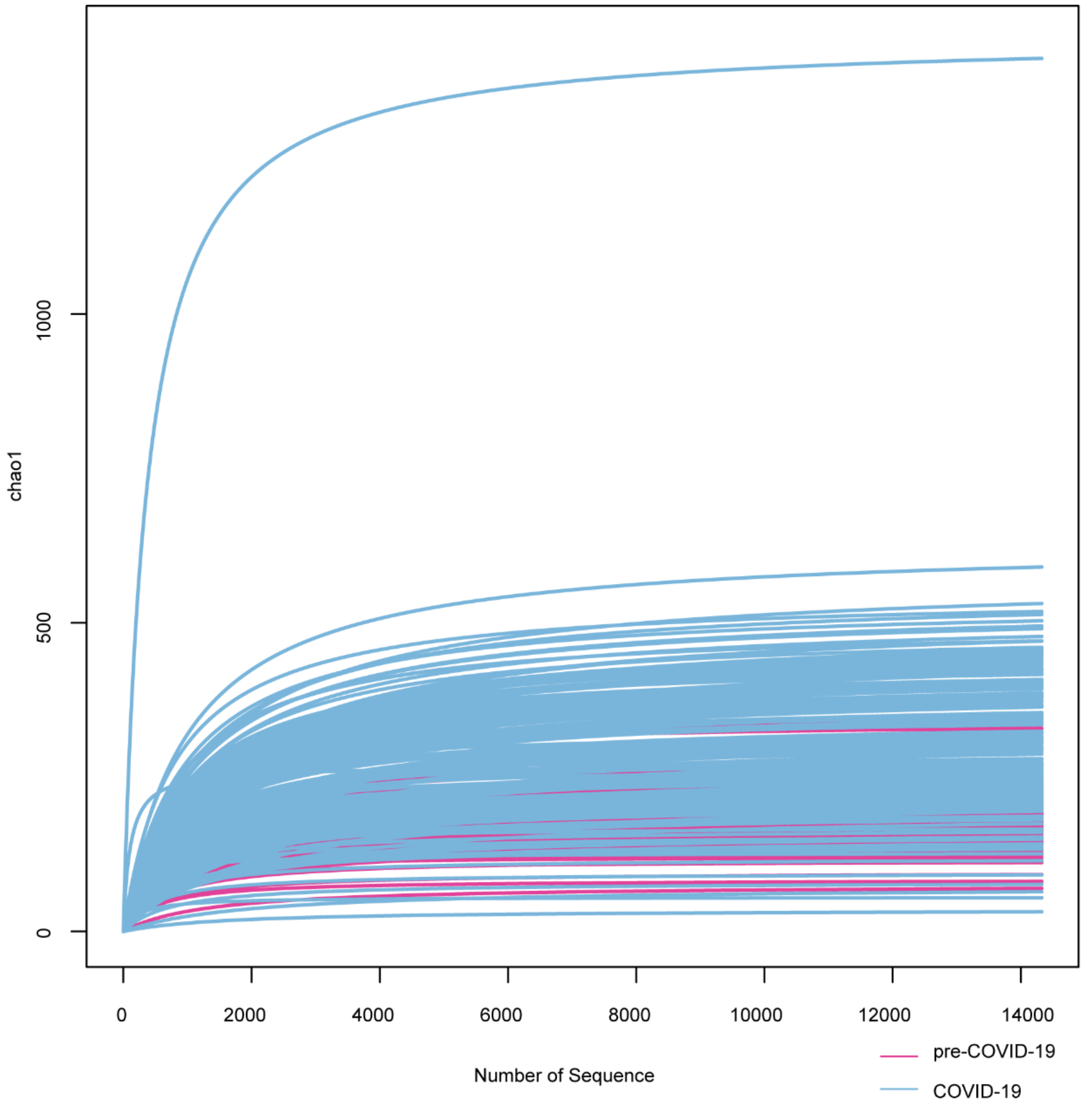

Figure S1. Rarefaction curves of 16S rRNA gene sequencing depth in the RSV-positive subgroup.

Rarefaction curves were generated for each sample based on the Chao1 index. The curves approached a clear plateau, indicating that the sequencing depth was sufficient to capture the majority of bacterial diversity across samples.
